# Linkage to HIV Care and Antiretroviral Therapy in Cape Town, South Africa

**DOI:** 10.1371/journal.pone.0013801

**Published:** 2010-11-02

**Authors:** Katharina Kranzer, Jennifer Zeinecker, Philip Ginsberg, Catherine Orrell, Nosindiso N. Kalawe, Stephen D. Lawn, Linda-Gail Bekker, Robin Wood

**Affiliations:** 1 Department of Medicine, Faculty of Health Sciences, The Desmond Tutu HIV Centre, Institute for Infectious Disease and Molecular Medicine, University of Cape Town, Cape Town, South Africa; 2 Clinical Research Unit, Department of Infectious and Tropical Diseases, London School of Hygiene and Tropical Medicine, London, United Kingdom; 3 False Bay Hospital, Cape Town, South Africa; The George Washington University Medical Center, United States of America

## Abstract

**Background:**

Antiretroviral therapy (ART) has been scaled-up rapidly in Africa. Programme reports typically focus on loss to follow-up and mortality among patients receiving ART. However, little is known about linkage and retention in care of individuals prior to starting ART.

**Methodology:**

Data on adult residents from a periurban community in Cape Town were collected at a primary care clinic and hospital. HIV testing registers, CD4 count results provided by the National Health Laboratory System and ART registers were linked. A random sample (n = 885) was drawn from adults testing HIV positive through antenatal care, sexual transmitted disease and voluntary testing and counseling services between January 2004 and March 2009. All adults (n = 103) testing HIV positive through TB services during the same time period were also included in the study. Linkage to HIV care was defined as attending for a CD4 count measurement within 6 months of HIV diagnosis. Linkage to ART care was defined as initiating ART within 6 months of HIV diagnosis in individuals with a CD4 count ≤200 cells/µl taken within 6 months of HIV diagnosis.

**Findings:**

Only 62.6% of individuals attended for a CD4 count measurement within 6 months of testing HIV positive. Individuals testing through sexually transmitted infection services had the best (84.1%) and individuals testing on their own initiative (53.5%) the worst linkage to HIV care. One third of individuals with timely CD4 counts were eligible for ART and 66.7% of those were successfully linked to ART care. Linkage to ART care was highest among antenatal care clients. Among individuals not yet eligible for ART only 46.3% had a repeat CD4 count. Linkage to HIV care improved in patients tested in more recent calendar period.

**Conclusion:**

Linkage to HIV and ART care was low in this poor peri-urban community despite free services available within close proximity. More efforts are needed to link VCT scale-up to subsequent care.

## Introduction

South Africa is home to one-sixth of the world's population living with HIV and has the largest antiretroviral therapy (ART) programme in the world [1], [2]. ART roll out began nationally in late 2003 and by the middle of 2008, 568,000 adults and children were receiving ART. This translated into around 40% of eligible adults receiving ART in 2008 [3], although the latest guidelines recommend earlier initiation for certain patients, thus increasing the numbers eligible for ART and widening the treatment gap [4].

In an effort to increase access to prevention and care, South Africa launched an ambitious national campaign in April 2010 aiming to test 15 million people for HIV and to reach 1.5 million people with ART by June 2011. Increased HIV testing may impact on risk behavior in the short-term [5]. However, there is also a need to ensure that those who need treatment are linked to the appropriate services while those not eligible for treatment are monitored and started on ART when appropriate. A study from Durban, South Africa, reported that almost two-thirds of newly diagnosed patients accessing care in a semi-private hospital were lost to care between HIV diagnosis and getting a CD4 count, and another one in five patients were lost between CD4 testing and ART initiation [6], [7]. Another study from South Africa found that only 45% of eligible patients started ART in a public sector ART project in Free State. Mortality and TB incidence in patients failing to initiate ART was more than 2 times higher compared to patients initiating ART [8].

The impact of ART on mortality, morbidity, TB incidence [9], [10] and HIV transmission [11] at a population level depends on ART coverage. ART coverage defined as the number of patients receiving ART at a point in time, divided by the number needing treatment is determined by timely HIV diagnosis and effective linkage to ART. This study investigates linkage to HIV and ART care using a random sample of individuals testing HIV positive either provided-initiated (through antenatal care (ANC), tuberculosis (TB), sexually transmitted infection (STI) services) or client-initiated (through voluntary counseling and testing (VCT) services) in a peri-urban township in the Western Cape Province in South Africa. Linkage to care was defined first as attending for a CD4 count measurement within 6 months of a positive HIV test and second as the proportion of eligible individuals starting ART within 6 months of their HIV diagnosis.

## Methods

### Setting

The study was based in a peri-urban township in the greater area of Cape Town, with a population of approximately 15,000 people and a measured adult HIV prevalence of 23% in 2005 [12]. The community is served by a single public-sector primary care clinic, which provides outpatient care including ART free of charge. A nearby hospital (5 km away) provides all secondary care for the population, including inpatient and antenatal services. The hospital also provides ART for some HIV-infected individuals from the community.

### HIV testing, CD4 count measurements and ART services

Client-initiated HIV testing services have been available to all individuals accessing either the local clinic or the hospital since 2001. Clients who tested on their own initiative are referred to as having tested through VCT services. Provider-initiated testing was routinely provided to any patient accessing TB services whose HIV status was unknown. This was extended to all pregnant females accessing the hospital or clinic in 2002 and patients accessing STI services in 2007. All testing required signed consent. All CD4 count tests were free for patients and performed by the centrally located National Health Laboratory Services (NHLS) in Cape Town.

ART provision at the primary health care clinic and hospital began in 2004.

### Linkage to HIV and ART care

Linkage to HIV care was defined as attending for a CD4 count measurement within 6 months of HIV diagnosis. We did not ascertain if individuals actually received their CD4 counts. Linkage to ART care was defined as initiating ART within 6 months of HIV diagnosis in individuals with a CD4 count ≤200 cells/µl taken within 6 months of HIV diagnosis. Having a repeat CD4 count was defined as having had a repeated CD4 count in individuals not yet eligible for ART (CD4 count >200 cells/µl) and tested before 2009.

### Data collection

We collected data from 3 sources. First, the primary care clinic and hospital HIV testing registers provided all data on HIV infected, adult community residents (≥18 years) diagnosed between January 2004 and March 2009. Data at the primary health care clinic were missing for the period from February 2008 to August 2008. For each test encounter recorded in the registers, we retrieved data on client identification variables (first name, surname, date of birth, and medical record number); place of residence; sex; test acceptance; test result and service. For HIV infected individuals who tested more than once, the earliest positive HIV test was considered. Second, data on CD4 counts performed at either the primary care clinic or the hospital in the period from 2004 to October 2009 were obtained from NHLS. The date of CD4 count was the date the client provided blood. Third, data from residents who initiated ART care at the primary health care clinic or hospital were obtained from electronic ART registers at the clinic and hospital.

These three databases were merged on first name, surname, medical record number and date of birth. In cases where identifiers did not match completely two researchers (PG and KK) independently confirmed that records in different databases were from the same individual. Concordance between the two researchers was 97%. Cases where the two researchers disagreed were discussed until consensus was reached. For all subsequent analysis data was stripped of all personal identifiers.

### Ethics

Written informed consent was obtained from all individuals initiated on ART and screened for ART. Individuals testing for HIV are routinely entered into the HIV testing register. Informed consent was not obtained from HIV positive individuals not linking to care, as this was a retrospective study and individuals were not actively follow-up. Data collection and analysis was approved by the University of Cape Town Ethics Committee and Partners Human Subjects Institutional Review Board and the London School of Hygiene and Tropical Medicine.

### Statistical analysis

A random sample (n = 885) of adults testing HIV positive through ANC, STI and VCT services between January 2004 and March 2009 was selected for this analysis. All adults testing positive through TB services were included in this analysis to ensure an adequate sample size in this group.

All analyses were carried out using Stata version 11 (Stata Corp. LP, College Station, TX, United States of America). Proportions were calculated stratified by service. Total proportions were calculated taking the different sampling proportions into account. Risk ratios investigating associations between age, sex, calendar period and timely linkage to HIV care, CD4 count ≤200 cells/µl and repeated CD4 counts were estimated for each service. Risk ratios were calculated using a log binominal model [13].

## Results

### HIV testing and HIV prevalence

A total of 8515 records of HIV tests were available for adult members of the community. The majority of individuals tested through VCT (n = 5345, 62.8%) services (Table 1). The overall HIV prevalence among those tested was 23.5% with the highest prevalence among patients tested through TB (37.9%) and VCT services (24.9%) (χ^2^ test, p<0.01). The median age of individuals tested was 26 (interquartile range (IQR), 22–32) and 67.9% were women. HIV prevalence was 21.6% in men and 24.4% in women.

**Table 1:** Number (%) of individuals who tested for HIV and who were found to be positive stratified by type of clinical service.

	ANC service	STI service	TB service	VCT service	Total
**Tested N (%)**	1525 (17.9)	1370 (16.1)	275 (3.2)	5345 (62.8)	8515 (100)
**Positives N (%)**	332 (16.6)	237 (11.8)	103 (5.1)	1330 (66.4)	2002 (100)
**HIV Prevalence**	21.8%	17.3%	37.5%	24.9%	23.5%

All HIV testing records available for the period from January 2004 until March 2009 from adult patients were included in this analysis.

ANC = antenatal care, STI = sexual transmitted infections, TB = tuberculosis, VCT = voluntary counseling and testing.

A total of 2002 clients tested HIV positive. Their median age was 28 years (IQR, 24–33) and the majority were women (70.3%). The proportion of women testing HIV positive was 100% in ANC, 66.2% in STI, 38.8% in TB and 66.4% in VCT clients. 1330 (66.4%) individuals tested HIV positive through VCT, 332 (16.6%) through ANC, 237 (11.8%) through STI and 103 (5.1%) through TB services.

### Linkage to HIV and ART care

Linkage to HIV and ART care was assessed in a random sample of 47% of individuals testing HIV positive through ANC, STI and VCT services and 100% of individuals testing through TB services: 150 tested through ANC, 113 through STI, 662 through VCT and 103 through TB services. Only 62.6% (95%CI 59.6–65.5) of clients attended for a CD4 count measurement within 6 months of testing HIV positive (Table 2) and 26.3% (95%CI 23.5–29.0) did not have any recorded CD4 count test. The proportion of individuals attending for a CD4 count measurement within 6 months was highest among individuals tested through ANC (81.3%) and STI (84.1%) services and lowest among those who learnt of their status via VCT (53.5%) (Table 2).

**Table 2:** Percentage of individuals linking to HIV care (as defined by attending for a CD4 cell count measurement), distribution of CD4 count measurements, percentage of patients subsequently initiating ART and percentage of clients non-eligible for ART returning for a repeat CD4 count.

Variables		ANC (n = 150)% (N)	STI (n = 113)% (N)	TB (n = 103)% (N)	VCT (n = 622)% (N)	Total%
**First CD4 count after HIV test**	**≤6 months**	81.3 (122)	84.1 (95)	68.9 (71)	53.5 (333)	62.6 (59.6–65.5)
	**>6 months**	2.0 (3)	2.7 (3)	13.6 (14)	14.8 (92)	11.1 (9.2–13.1)
	**None**	16.7 (25)	13.3 (15)	17.5 (18)	31.7 (197)	26.3 (23.5–29.0)
**First CD4 count within 6months of HIV test**	**≤200 cells/µl**	14.8 (18)	22.1 (21)	54.9 (39)	42.3 (141)	34.1 (30.4–37.7)
	**201–350 cells/µl**	24.6 (30)	32.6 (31)	23.9 (17)	23.7 (79)	25.3 (21.8–28.8)
	**>351 cells/µl**	60.7 (74)	45.3 (43)	21.1 (15)	33.9 (113)	40.6 (36.8–44.4)
**ART initiation within 6 months of HIV test in eligible individuals with timely first CD4 count**	**Yes**	72.2 (13)	52.4 (11)	71.8 (28)	67.4 (95)	66.7 (60.2–73.1)
	**No**	27.8 (5)	47.6 (10)	28.2 (11)	32.6 (46)	33.3 (26.9–39.*)
**Repeat CD4 count in individuals with a first CD4 count >200 cells/µl**	**Yes**	48.5 (47)	57.6 (38)	34.5 (10)	42.3 (96)	46.3 (41.4–51.1)
	**No**	51.5 (50)	42.4 (28)	65.5 (19)	57.7 (131)	53.7 (48.9–58.6)

ANC = antenatal care, STI = sexual transmitted infections, TB = tuberculosis, VCT = voluntary counseling and testing.

Among individuals with a CD4 count measurement within 6 months, 34.1% (95%CI 30.4–37.7) were eligible for ART according the South African Department of Health criteria (CD4 count ≤200 cells/µl) at the time of the study (Table 2). Low CD4 counts were more prevalent among individuals tested through TB (54.9%) and VCT services (42.6%). In individuals attending for a CD4 count measurement within 6 months the median time between HIV test and CD4 count measurement was: 2 days (IQR 2–6) for ANC, 3 days (IQR 2–4) for STI, 3 days (IQR 2–5) for TB and 2 days (IQR 2–4) for VCT clients. Overall 4.3% of clients attended for a CD4 test at the same day as the HIV test. The majority of clients attended for CD4 count testing within 1 week (84.9%), 14.2% within 8 days and 3 months and only 0.9% within 3 and 6 months.

In individuals with a delayed first CD4 count measurements, the mean time between HIV diagnosis and first CD4 count was 490 days (IQR 345–769). Among patients with delayed first CD4 count measurements, 33.2% (95%CI 24.3–42.1) had a CD4 count ≤200 cells/µl and 26.2% (95%CI 17.8–34.8)−43.2) had a CD4 count of 201–350 cells/µl.

Only 66.7% (95% CI 60.2–73.1) of eligible individuals with a timely CD4 count accessed ART care within 6 months of HIV testing (Table 2). Linkage to ART care was highest among individuals tested through ANC services (72.2%). Among individuals not yet eligible for ART only 46.3% (95%CI 41.4–51.1) ever had a repeat CD4 count. Median time between the first and the second CD4 count was 236 days.

**Figure 1:**
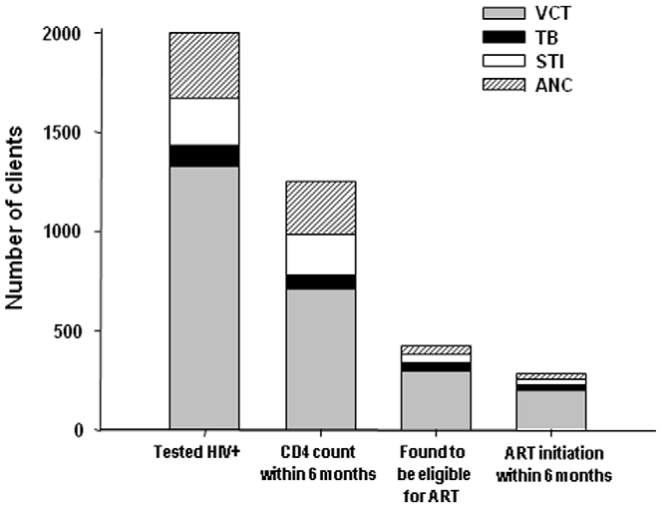
Number of clients testing HIV+, with timely CD4 counts, eligible for ART and initiating ART estimated using proportions from **table 2**. ART = antiretroviral therapy, ANC = antenatal care, STI = sexual transmitted infections, TB = tuberculosis, VCT = voluntary counseling and testing.

Figure 1 summarizes the number of people tested through different services and the numbers linking to HIV and ART care by service using the proportions estimated from the random sample.

### Predictors of low CD4 count, linkage to HIV, and repeated CD4 counts

Risk ratios investigating predictors for linkage to HIV care showed that linkage to care in TB (RR 1.67, 95%CI 1.27–2.21) and VCT (RR 1.60, 95%CI 1.40–1.84) clients was more likely in 2007–2009 compared to 2004–2006 (table 3). This was not the case for ANC (RR 0.97) clients who were slightly less likely to link to HIV care if tested more recently (table 3). Linkage to ART care could only be assessed in VCT clients due to the small sample size in the other groups. Neither age (RR 0.85, 95%CI 0.67–1.09) nor sex (RR 1.03, 95% CI 0.81–1.31) nor year of testing (RR 1.02, 95% CI 0.81–1.30) predicted linkage to HIV care in VCT clients.

**Table 3:** Factors associated with linkage to HIV care (attending for a CD4 count measurement within 6 months of HIV diagnosis) stratified by service.

Variables	ANC	STI	TB	VCT
	RR (95% CI)	RR (95% CI)	RR (95% CI)	RR (95% CI)
**Female**	NA	1	1	1
**Male**	NA	0.93 (0.79–1.09)	1.01 (0.82–1.25)	1.10 (1.01–1.33)
**Age<30 years**	1	1	1	1
**Age≥30 years**	0.97 (0.90–1.04)	1.17 (1.01–1.35)	1.07 (0.84–1.35)	1.16 (0.96–1.26)
**Tested in 2004–2006**	1	NA	1	1
**Tested in 2007–2009**	**0.87 (0.74–0.99)**	NA	**1.67 (1.27–2.21)**	**1.60 (1.40–1.84)**

ANC = antenatal care, STI = sexual transmitted infections, TB = tuberculosis, VCT = voluntary counseling and testing.

NA = not applicaple.

The risk of having CD4 count measurement ≤200 cells/µl was higher in individuals aged more than 30 years regardless which service they tested through (table 4). Repeated CD4 counts were 1.3 times more likely in individuals more than 30 years of age, but this result only reached significance in the VCT clients (RR 1.25, 95% CI 1.00–1.55).

**Table 4:** Factors associated with having a CD4 count ≤200 cells/µl within 6 months of HIV diagnosis.

Variables	ANC	STI	Tb	VCT
	RR (95% CI)	RR (95% CI)	RR (95% CI)	RR (95% CI)
**Female**	NA	1	1	1
**Male**	NA	0.82 (0.36–1.86)	0.97 (0.62–1.50)	1.27 (0.99–1.63)
**Age<30 years**	1	1	1	1
**Age≥30 years**	**2.42 (1.03–5.68)**	2.00 (0.92–4.35)	1.10 (0.68–1.78)	**1.40 (1.07–1.82)**
**Tested in 2004–2006**	1	NA	1	1
**Tested in 2007–2009**	1.32 (0.57–3.08)	NA	1.18 (0.75–1.85)	0.94 (0.74–1.19)

ANC = antenatal care, STI = sexual transmitted infections, TB = tuberculosis, VCT = voluntary counseling and testing.

## Discussion

This study evaluated the proportion of individuals linking to HIV care in a public sector service in Cape Town, South Africa. Only 63% of patients attended for a CD4 count measurement within 6 months of diagnosis. Although a substantial proportion of patients had CD4 counts ≤200 cells/µl (34%) and were therefore eligible for ART according to South African guidelines [14], only 67% of these started ART within 6 months. Among those who did have a timely CD4 count but were not yet eligible for ART, only 46% returned for a repeat CD4 count after a median time of 8 months. Individuals testing through ANC services had better linkage to HIV and ART care and higher CD4 counts at time of HIV diagnosis compared to individuals accessing the other services.

HIV is a chronic disease and comprehensive HIV care needs to be provided within a continuum of care [15]. ART is just one of the components of HIV care and care of individuals not yet requiring ART is equally important [16]. The continuum of HIV care starts when an individual is diagnosed with HIV. ART eligibility should be assessed when individuals are newly diagnosed and in regular (6 monthly) intervals thereafter. Individuals not yet eligible for ART should receive comprehensive HIV care including cotrimoxazole, isoniazid preventive therapy, screening for TB and cervical cancer, contraceptive advice, counseling and social support until they become eligible for ART. Following initiation of ART individuals needs to be supported within the same framework to ensure good adherence and retention in care.

We identified a number of important issues in our study. First, people who tested on their own initiative were least likely to have a timely CD4 count measurement done, underscoring the need to ensure that scale up of VCT programmes will be accompanied by clear plans to ensure that those who test positive go on to receive appropriate care. Second, almost a third (28%) of eligible patients with TB did not receive ART despite recommendations in favour of concomitant treatment since 2003 [17], and ART being associated with a 64–95% reduction in mortality in such patients [10], [18], [19], [20]. This underscores the importance of integrating HIV and TB services [21].

This study shows that men and younger adults fail to access health services efficiently. Only 30% of clients tested for HIV were men. This is consistent with studies showing that HIV-infected men are less likely to access treatment [22], [23], present with more advanced stages of HIV disease [24] and have a higher mortality risk during ART [25], [26], [27], [28], [29], [30], [31], [32]. Repeated CD4 counts were less likely in individuals under 30 years of age as also reported elsewhere[33].

It is important to note that less than half of patients whose first CD4 count was above the ART eligibility threshold came back for a repeat test. One way of improving ART uptake, and thus reduce mortality among patients who are otherwise lost to care, might be to change the CD4 threshold to 350 cell/µl in line with the latest World Health Organization recommendations [34].

Our overall finding that 33% of patients eligible for ART were lost to care is consistent with several reports from elsewhere in southern Africa. In a programme report from South Africa, only 55% of patients had a CD4 count measurement within 8 weeks of HIV diagnosis and 81% of eligible patients were on ART at 3 months follow-up [6], [7]. Out of 2483 patients eligible for ART in Uganda 637 (26%) did not start ART; a third of these patients died before ART initiation and another quarter were alive but not taking ART [25]. In Mozambique only 57% of patients testing HIV positive entered HIV care and 31% of patients eligible for ART started ART within 3 months [35].

In our study only 63% of patients testing positive for HIV attended for a CD4 count measurement within 6 months. These outcomes are worse than those recently reported by a public-sector clinic in Johannesburg where 84.6% of patients who tested positive for HIV had a CD4 count measurement. The majority of these patients did not return for their CD4 result within 12 weeks [36]. Data from the same clinic in Johannesburg showed that among patients not yet eligible for ART only 26% returned for a scheduled pre-ART medical visit within one year compared to 43% of our patients not yet eligible for ART returning for a repeat CD4 count [37].

Substantial improvement in linkage to HIV care for TB and VCT patients was observed in more recent years in this study and yet this was not accompanied by improvements in linkage to ART. Failure of linkage to HIV and ART services translates into incomplete ART coverage at population level, seriously undermining the potential for reductions in mortality, morbidity, TB incidence and HIV transmission.

The study has several strengths and limitations. Strengths include that the study was conducted in a routine clinical program where CD4 count testing and ART were provided free. Thus, the results should be generalisable to similar settings. The study was conducted over a prolonged period with increasing ART availability. Among the limitations is the fact that patients might have been misclassified as failing to link to care if they accessed care with a service provider other than the primary health care clinic or hospital. Thus, linkage to care might be underestimated. However the nearest other ART site is more than 10 km away, and residents of this poor community are unlikely to have sought care in such a distant ART site unless they had moved away. Second, we did not assess if patients who had a CD4 count measurement actually returned to receive the result. Thirdly, we did not investigate reasons for not linking to care. Studies that have ascertained outcomes among patients lost to care have reported that up to a third of patients who failed to initiate ART had died [7], [8], [25]. Time cut-offs for linkage to care for both timely CD4 count and ART initiation are somewhat arbitrary. When no time cut-offs were used 75.3% (95% CI 70.3–80.3) of eligible individuals who had a CD4 count at some point during the study period eventually initiated ART.

In conclusion, while considerable attention has been paid to loss to follow-up and mortality among patients receiving ART [32], [38], [39], [40], data on losses at earlier stages of the care pathway are scarce. As our study shows, a focus only on outcomes of those patients fortunate enough to initiate treatment fails to account for a substantial number of patients who are eligible for ART but do not receive it or not yet eligible but fail to reappear. Pre-ART defaulting should be encouraged in programme reporting. Programme adaptation to ensure retention in care between testing and ART should consider point of care CD4 count testing at time of HIV diagnosis as well as provision of integrated TB and HIV.
